# Branched-chain amino acid metabolism is regulated by ERRα in primary human myotubes and is further impaired by glucose loading in type 2 diabetes

**DOI:** 10.1007/s00125-021-05481-9

**Published:** 2021-06-16

**Authors:** Rasmus J. O. Sjögren, David Rizo-Roca, Alexander V. Chibalin, Elin Chorell, Regula Furrer, Shintaro Katayama, Jun Harada, Håkan K. R. Karlsson, Christoph Handschin, Thomas Moritz, Anna Krook, Erik Näslund, Juleen R. Zierath

**Affiliations:** 1 Department of Molecular Medicine and Surgery, Integrative Physiology, Karolinska Institutet, Stockholm, Sweden; 2 Department of Public Health and Clinical Medicine, Umeå University, Umeå, Sweden; 3 Biozentrum, University of Basel, Basel, Switzerland; 4 Department of Biosciences and Nutrition, Karolinska Institutet, Huddinge, Sweden; 5 Cardiovascular-Metabolics Research Laboratories, Daiichi Sankyo Co., Ltd., Tokyo, Japan; 6 Swedish Metabolomics Centre, Department of Forest Genetics and Plant Physiology, Swedish University of Agricultural Sciences, Umeå, Sweden; 7 Department of Physiology and Pharmacology, Integrative Physiology, Karolinska Institutet, Stockholm, Sweden; 8 Division of Surgery, Department of Clinical Sciences, Danderyd Hospital, Karolinska Institutet, Stockholm, Sweden

**Keywords:** Branched-chain amino acid, Oestrogen-related receptor α, Oral glucose tolerance test, Peroxisome proliferator-activated receptor γ coactivator 1-α, Skeletal muscle, Type 2 diabetes

## Abstract

**Aims/hypothesis:**

Increased levels of branched-chain amino acids (BCAAs) are associated with type 2 diabetes pathogenesis. However, most metabolomic studies are limited to an analysis of plasma metabolites under fasting conditions, rather than the dynamic shift in response to a metabolic challenge. Moreover, metabolomic profiles of peripheral tissues involved in glucose homeostasis are scarce and the transcriptomic regulation of genes involved in BCAA catabolism is partially unknown. This study aimed to identify differences in circulating and skeletal muscle BCAA levels in response to an OGTT in individuals with normal glucose tolerance (NGT) or type 2 diabetes. Additionally, transcription factors involved in the regulation of the BCAA gene set were identified.

**Methods:**

Plasma and vastus lateralis muscle biopsies were obtained from individuals with NGT or type 2 diabetes before and after an OGTT. Plasma and quadriceps muscles were harvested from skeletal muscle-specific *Ppargc1a* knockout and transgenic mice. BCAA-related metabolites and genes were assessed by LC-MS/MS and quantitative RT-PCR, respectively. Small interfering RNA and adenovirus-mediated overexpression techniques were used in primary human skeletal muscle cells to study the role of *PPARGC1A* and *ESRRA* in the expression of the BCAA gene set. Radiolabelled leucine was used to analyse the impact of oestrogen-related receptor α (ERRα) knockdown on leucine oxidation.

**Results:**

Impairments in BCAA catabolism in people with type 2 diabetes under fasting conditions were exacerbated after a glucose load. Branched-chain keto acids were reduced 37–56% after an OGTT in the NGT group, whereas no changes were detected in individuals with type 2 diabetes. These changes were concomitant with a stronger correlation with glucose homeostasis biomarkers and downregulated expression of branched-chain amino acid transaminase 2, branched-chain keto acid dehydrogenase complex subunits and 69% of downstream BCAA-related genes in skeletal muscle. In primary human myotubes overexpressing peroxisome proliferator-activated receptor γ coactivator-1α (PGC-1α, encoded by *PPARGC1A*), 61% of the analysed BCAA genes were upregulated, while 67% were downregulated in the quadriceps of skeletal muscle-specific *Ppargc1a* knockout mice. *ESRRA* (encoding ERRα) silencing completely abrogated the PGC-1α-induced upregulation of BCAA-related genes in primary human myotubes.

**Conclusions/interpretation:**

Metabolic inflexibility in type 2 diabetes impacts BCAA homeostasis and attenuates the decrease in circulating and skeletal muscle BCAA-related metabolites after a glucose challenge. Transcriptional regulation of BCAA genes in primary human myotubes via PGC-1α is ERRα-dependent.

**Graphical abstract:**

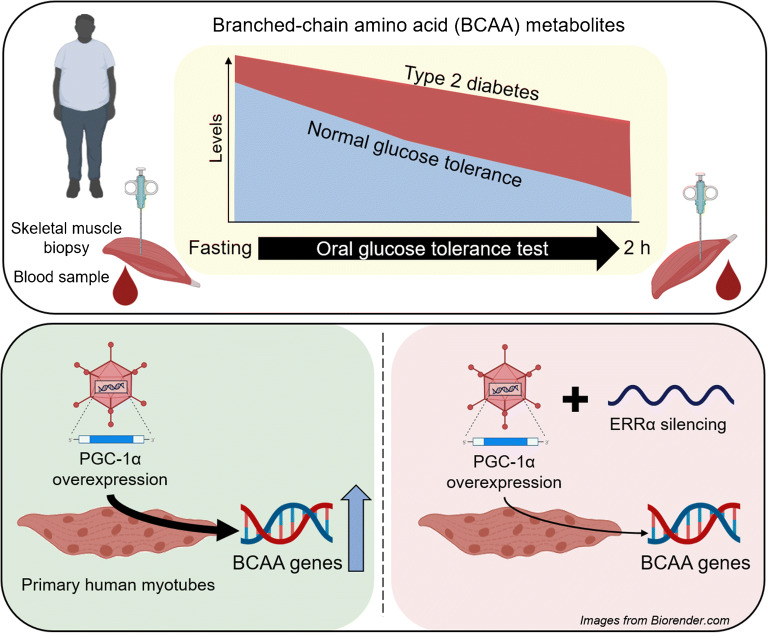

**Supplementary Information:**

The online version contains peer-reviewed but unedited supplementary material available at 10.1007/s00125-021-05481-9.

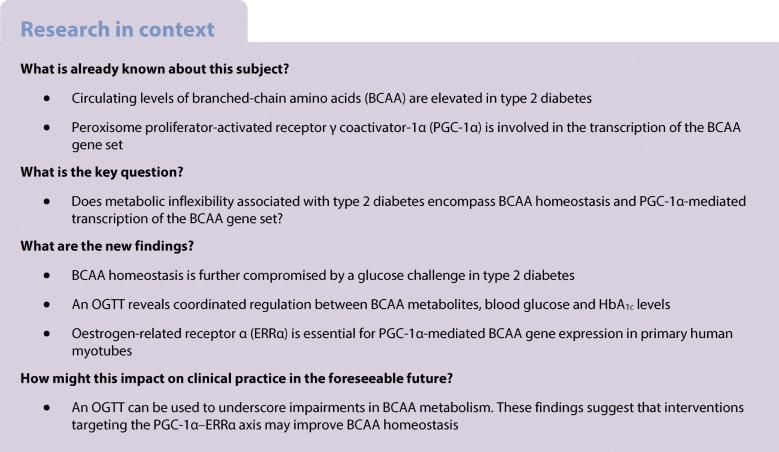



## Introduction

Type 2 diabetes is a metabolic disease characterised by chronic hyperglycaemia and insulin resistance [1]. These metabolic derangements severely affect pathways controlling the appropriate sensing and handling of nutrients, thereby leading to a positive energy balance and metabolic inflexibility, which further compromises whole-body glucose homeostasis [2]. Overnutrition and type 2 diabetes-related disturbances also affect non-glucose metabolites such as branched-chain amino acids (BCAAs; leucine, isoleucine and valine) [3]; the utilisation and metabolism of these essential amino acids are exquisitely regulated in healthy individuals. While BCAAs and related metabolites play a role in protein synthesis, they also modulate liver gluconeogenesis and lipogenesis rates, cell growth and nutrient signalling, and can enter the tricarboxylic acid cycle to produce energy [4]. Under pathological conditions, BCAAs, especially in a context of overnutrition, disrupt insulin sensitivity and secretion [5].

Circulating levels of BCAAs are elevated in individuals with obesity, insulin resistance and/or type 2 diabetes [6]. Metabolomic profiling of blood metabolites has revealed a signature of altered BCAA catabolism in obese individuals, strongly associated with insulin resistance [7]. BCAA-related metabolites are predictive of type 2 diabetes pathogenesis [8] as well as being prognostic markers for intervention outcomes [9, 10]. Mendelian randomisation analysis suggested a causal link between genetic variants associated with impaired BCAA catabolism and higher risk of type 2 diabetes [11]. Therefore, there is growing evidence that high levels of BCAAs and related intermediate metabolites are not only type 2 diabetes biomarkers but also pathophysiological factors. However, many of these studies were conducted in individuals after an overnight fast, under conditions in which protein degradation in skeletal muscle and a concomitant release of amino acids to support gluconeogenesis [12] could mask deeper alterations in BCAA homeostasis. Moreover, these studies do not provide insight into the dynamic shift in metabolism that occurs in response to nutritional challenges. An analysis of metabolomic signatures in fasted individuals with glucose tolerance or type 2 diabetes before and after a glucose challenge may provide insight into dynamic changes in BCAAs and related metabolites and the consequences of insulin resistance.

Skeletal muscle is the largest contributor to systemic BCAA oxidation [13] and therefore impairments in glucose and BCAA metabolism in myocytes have an impact on whole-body metabolic homeostasis. While BCAA-related gene expression and oxidation rates are reduced in vastus lateralis muscle from individuals with insulin resistance [14], studies of BCAA metabolism in human skeletal muscle are scarce. BCAA catabolism occurs in the mitochondrial matrix, implying that alterations in mitochondrial proteins may influence BCAA metabolism. In transgenic mice overexpressing the mitochondrial biogenesis inducer peroxisome proliferator-activated receptor γ (PPARγ) coactivator-1α (PGC-1α), BCAA levels are reduced in gastrocnemius muscle [15]. Conversely, administration of the PPARγ agonist thiazolidinedione rosiglitazone improves glycaemic control and increases circulating levels of BCAAs in individuals with type 2 diabetes [16]. Nevertheless, mechanisms underlying the direct role of PGC-1α in the regulation of BCAA catabolism are unclear.

The aim of this study was to assess whether an OGTT can further reveal an impairment in BCAA metabolism in individuals with type 2 diabetes. We also aimed to identify a PGC-1α transcriptional partner in the regulation of the BCAA gene program by using cell and mouse models in which PGC-1α expression was altered.

## Methods

### Participants

**Fig. 1:**
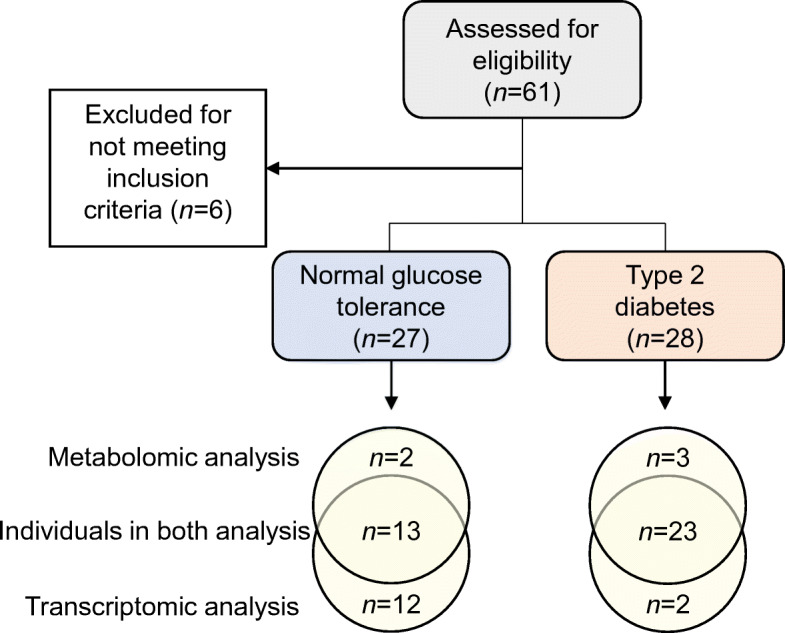
Flow chart illustrating participant enrolment and analysis. Sixty-one individuals were assessed for eligibility and six were excluded for not meeting the inclusion criteria. Group size for transcriptomic and metabolomic analysis is reported. Transcriptomic and metabolomic analysis shared samples from 13 individuals with NGT and 23 with type 2 diabetes

Thirty-two men with normal glucose tolerance (NGT) and 29 men with type 2 diabetes aged 44–69 years were recruited to participate in this study. All participants gave their informed consent. The study was approved by the regional ethical review board in Stockholm. The experimental procedures were conducted according to the Declaration of Helsinki. Participants underwent a clinical health screening consisting of clinical chemistry and anthropometric measurements (Tables 1, 2). Five individuals in the control group that exhibited impaired glucose tolerance and one individual diagnosed with type 2 diabetes that exhibited NGT were excluded from the study (Fig. 1). Individuals with type 2 diabetes had higher blood glucose, HbA_1c_ and HOMA-IR, as well as higher BMI and waist circumference than individuals with NGT (Tables 1 and 2). Individuals with type 2 diabetes included in the transcriptomic analysis were older than the control group of individuals with NGT (Table 2). Individuals with type 2 diabetes were treated with metformin (*n* = 25; daily dose range 500–3000 mg) and/or sulfonylurea (glibenclamide [glyburide], *n* = 5, 2–5 mg daily; glimepiride, *n* = 1, 3 mg daily; glipizide, *n* = 1, 5 mg daily). Glucose-lowering medication was taken by the individuals with type 2 diabetes after the skeletal muscle biopsy collection.

**Table 1:** Clinical characteristics of the participants included in the metabolomic analysis

Characteristic	NGT	Type 2 diabetes	*p* value
Age (years)	59 ± 2.4	62 ± 1.2	0.1611
Weight (kg)	81 ± 2.7	88 ± 1.8	0.0209
Height (m)	1.77 ± 0.02	1.79 ± 0.01	0.4082
BMI (kg/m^2^)	25.95 ± 0.59	27.66 ± 0.47	0.0307
Waist (cm)	90 ± 1.6	100 ± 1.6	0.0002
Hip (cm)	97 ± 1.2	101 ± 1.2	0.0241
WHR	0.93 ± 0.01	0.99 ± 0.01	0.0008
Pulse (beats/min)	62 ± 2.0	68 ± 1.6	0.0234
Fasting plasma glucose (mmol/l)	5.41 ± 0.13	8.59 ± 0.37	<0.0001
120 min plasma glucose (mmol/l)	6.05 ± 0.25	16.66 ± 0.58	<0.0001
HbA_1c_ (mmol/mol)	36.13 ± 1.13	51.19 ± 1.32	<0.0001
HbA_1c_ (%)	5.5 ± 0.10	6.8 ± 0.12	<0.0001
Serum insulin (pmol/l)	52.37 ± 5.30	85.51 ± 9.06	0.0124
120 min serum insulin (pmol/l)	294.2 ± 43.41	327.5 ± 42.06	0.6155
Plasma creatinine (μmol/l)	84.53 ± 2.65	80.72 ± 2.17	0.2794
Plasma AST (μkat/l)	0.42 ± 0.04	0.39 ± 0.03	0.5992
Plasma ALT (μkat/l)	0.38 ± 0.06	0.48 ± 0.04	0.1161
Plasma triacylglycerol (mmol/l)	1.08 ± 0.14	1.41 ± 0.13	0.1093
Plasma cholesterol (mmol/l)	5.27 ± 0.17	4.50 ± 0.14	0.0014
Plasma HDL-cholesterol (mmol/l)	1.33 ± 0.06	1.24 ± 0.06	0.3562
Plasma LDL-cholesterol (mmol/l)	3.47 ± 0.13	2.62 ± 0.14	0.0003
Serum C-peptide (nmol/l)	0.69 ± 0.04	0.99 ± 0.08	0.0115
HOMA-IR	1.71 ± 0.22	5.28 ± 0.65	0.0013

Results are mean ± SEM for individuals with NGT (*n* = 15) and type 2 diabetes (*n* = 26)

Statistical analysis was performed using Student’s *t* test

ALT, alanine aminotransferase; AST, aspartate aminotransferase

**Table 2:** Clinical characteristics of the participants included in the transcriptomic analysis

Characteristic	NGT	Type 2 diabetes	*p* value
Age (years)	58 ± 1.8	62 ± 1.3	0.0185
Weight (kg)	84 ± 1.7	89 ± 1.9	0.0314
Height (m)	1.80 ± 0.02	1.79 ± 0.01	0.7185
BMI (kg/m^2^)	25.98 ± 0.35	27.94 ± 0.48	0.0020
Waist (cm)	93 ± 1.4	101 ± 1.7	0.0020
Hip (cm)	99 ± 0.9	101 ± 1.3	0.1691
WHR	0.94 ± 0.01	0.99 ± 0.01	0.0009
Pulse (beats/min)	61 ± 1.4	68 ± 1.6	0.0072
Fasting plasma glucose (mmol/l)	5.46 ± 0.08	8.56 ± 0.39	<0.0001
120 min plasma glucose (mmol/l)	5.89 ± 0.27	16.20 ± 0.69	<0.0001
HbA_1c_ (mmol/mol)	35.76 ± 0.64	50.44 ± 1.50	<0.0001
HbA_1c_ (%)	5.4 ± 0.06	6.8 ± 0.14	<0.0001
Serum insulin (pmol/l)	52.71 ± 5.58	92.58 ± 9.01	0.0008
120 min serum insulin (pmol/l)	314.9 ± 50.8	350.5 ± 42.4	0.5937
Plasma creatinine (μmol/l)	83.64 ± 2.36	79.71 ± 2.29	0.2307
Plasma AST (μkat/l)	0.38 ± 0.03	0.40 ± 0.03	0.7613
Plasma ALT (μkat/l)	0.42 ± 0.05	0.50 ± 0.04	0.2565
Plasma triacylglycerol (mmol/l)	1.25 ± 0.15	1.43 ± 0.13	0.4415
Plasma cholesterol (mmol/l)	5.40 ± 0.20	4.48 ± 0.15	0.0005
Plasma HDL (mmol/l)	1.30 ± 0.05	1.19 ± 0.06	0.1709
Plasma LDL (mmol/l)	3.56 ± 0.17	2.63 ± 0.15	0.0002
Serum C-peptide (nmol/l)	0.69 ± 0.04	1.03 ± 0.08	0.0008
HOMA-IR	1.86 ± 0.21	5.23 ± 0.64	<0.0001

Results are shown as mean ± SEM for individuals with NGT (*n* = 25) and type 2 diabetes (*n =* 25)

Statistical analysis was performed using Student’s *t* test

ALT, alanine aminotransferase; AST, aspartate aminotransferase

Participants arrived at the clinic at 07:45 h after a 12 h fast. A catheter was inserted into an arm vein and fasting blood samples were obtained. After applying local anaesthesia subcutaneously (lidocaine hydrochloride 5 mg/ml; Aspen Nordic, Denmark), biopsies from the vastus lateralis were obtained using a conchotome (AgnTho’s, Sweden). After ∼15 min, participants ingested a solution containing 75 g of glucose and underwent an OGTT. A blood sample was obtained 30 min after glucose ingestion. After 120 min, a blood sample and vastus lateralis biopsy were obtained. All biopsies were immediately snap-frozen in liquid nitrogen and stored at −80°C. Metabolomic analysis was performed by Metabolon (Durham, NC, USA) as described [17]. See electronic supplementary material (ESM) Methods: Human plasma and skeletal muscle metabolomics for details.

### Cell culture experiments

Human satellite cells were harvested from vastus lateralis skeletal muscle biopsies of healthy volunteers from both sexes and differentiated as described [18]. Mouse C2C12 myoblasts (ATCC CRL-1772; ATCC, VA, USA) were propagated in growth media (DMEM, 20% FBS and 1% antibiotic-antimycotic (11570486; Fisher Scientific, Sweden). After ~12 h, medium was changed to differentiation medium (DMEM, 2% horse serum and 1% antibiotic-antimycotic) and differentiated myotubes were cultured for 6 days before the final experiments. For pharmacological inhibition of oestrogen-related receptor α (ERRα), human skeletal muscle cells (HSMCs) were incubated with 5 μmol/l of XCT-790 (X4753; Sigma-Aldrich, Sweden), an inverse ERRα agonist, for 32 h. Cells were regularly tested to confirm the absence of mycoplasma contamination.

Small interfering RNAs (siRNAs) targeting *PPARGC1A* or *ESRRA* mRNA predesigned by ThermoFisher Scientific (Silencer Select; s21395 and s4830; Sweden) were used to silence the expression of these genes. Scramble siRNA (4390847; ThermoFisher Scientific) was used as negative control. Transfection of C2C12 myoblasts and HSMCs was performed 3 and 6 days after induction of differentiation, respectively, in OptiMEM reduced serum media (31985062; ThermoFisher Scientific) with Lipofectamine RNAiMAX (13778; ThermoFisher Scientific).

Adenoviral delivery of human and mouse *PPARGC1A* (Ad-PGC1A) or *gfp* (Ad-GFP) was performed overnight in differentiated HSMCs and C2C12 myotubes. Experiments were performed 48 h after inducing transduction. In cells that were both treated with siRNA and transduced, silencing was performed as described above and cells were transduced immediately after transfection.

### Mouse models

Male mice were housed under controlled lighting (12 h light–dark cycle) with free access to food and water. Experiments were performed in accordance with Swiss federal guidelines and were approved by the Kantonales Veterinäramt Basel-Stadt. Skeletal muscle-specific *Ppargc1a* transgenic mice (mTG) (C57BL/6-Tg(Ckm-*Ppargc1a*)31Brsp/J; The Jackson Laboratory, USA; https://www.jax.org/strain/008231) are described elsewhere [19]. The *Ppargc1a* muscle-specific knockout mice (mKO) were generated by breeding PGC-1α^loxP/loxP^ mice (B6N.129(FVB)-*Ppargc1a*^tm2.1Brsp^/J; The Jackson Laboratory; https://www.jax.org/strain/009666) [20] with transgenic mice expressing the Cre recombinase under the control of the human α-skeletal actin promoter (B6.Cg-Tg(ACTA1-cre)79Jme/J; The Jackson Laboratory; www.jax.org/strain/006149). Chow diet (AIN-93G; 7% fat, 58.5% carbohydrates, 18% protein and 16.5% of crude fibre, ash, and moisture) was provided by Provimi Kliba (Kaiseraugst, Switzerland).

Fasted (4 h) male mice aged 11–14 weeks were anaesthetised by intraperitoneal injection of pentobarbital (150 mg/kg) and tissues were collected. Metabolomic analysis of plasma and quadriceps muscle was performed at the Swedish Metabolomics Centre (Umeå University) as described [21]. See ESM Methods: Mouse plasma and skeletal muscle metabolomics for details.

### Quantitative real-time RT-PCR

Total RNA from human skeletal muscle biopsies, mouse skeletal muscles and cultured myotubes was extracted for mRNA analysis of BCAA genes. See ESM Methods: RNA isolation and relative mRNA expression for details. Primer sequences are shown in ESM Tables 1, 2.

### Western blot analysis

Equal amounts of protein from human skeletal muscle biopsies (10 μg) and cells (20 μg) were analysed for branched-chain α-keto acid dehydrogenase (BCKDHA), p^S293^-BCKDHA, branched-chain keto acid dehydrogenase kinase (BCKDK) and protein phosphatase, Mg^2+^/Mn^2+^-dependent 1K (PPM1K). See ESM Methods: Western blot analysis for details.

### Leucine oxidation

Myotube cultures were incubated with 1 ml of Ham’s F10 Medium in the presence of 0.1 mCi/ml radiolabelled leucine (l-[U-^14^C]leucine; 11.1 GBq/mmol, NEC279E250UC; PerkinElmer, MA, USA) and either 3,6-dichlorobenzo(b)thiophene-2-carboxylic acid (BT2) (ENA018104907; Sigma-Aldrich) or DMSO. Small cups were placed in cell-culture wells and plates were air-tight sealed. After incubation for 4 h at 37°C, 150 μl of 2 mol/l HCl and 300 μl of 2 mol/l NaOH were added into each well and small cup, respectively. After 1 h, the liberated CO_2_ was collected and subjected to scintillation counting (Tri-Carb 4910TR; PerkinElmer).

### Statistical analysis

Results in tables are reported as means ± SEM. Metabolites and gene expression results are reported as Tukey boxplots with median (line), 25–75% (box) and the 25th/75th percentile ±1.5 times the interquartile distance (whiskers). Values outside this range are plotted as individual open circles. Outlier values detected using the Grubb’s test are plotted as closed circles and were not considered in the statistical analysis. HSMCs from each donor, C2C12 myotubes at different passages, and single animals, were considered as experimental units. Randomisation and blinding were not carried out. Data normality was tested using the Shapiro–Wilk test. Homogeneity of variance was tested using the Levene’s test. Data that did not meet these criteria were transformed with Tukey’s ladder before the significance testing. Data were analysed using GraphPad Prism software (version 9.0.0; GraphPad Software, USA). Statistical tests and data information are indicated in the figure legends.

A network graph showing top-ranked proteins interacting with PGC-1α was generated using the STRING database (https://string-db.org/; accessed 18 May 2020) [22].

## Results

### Glucose loading further attenuates BCAA catabolism in individuals with type 2 diabetes

**Fig. 2:**
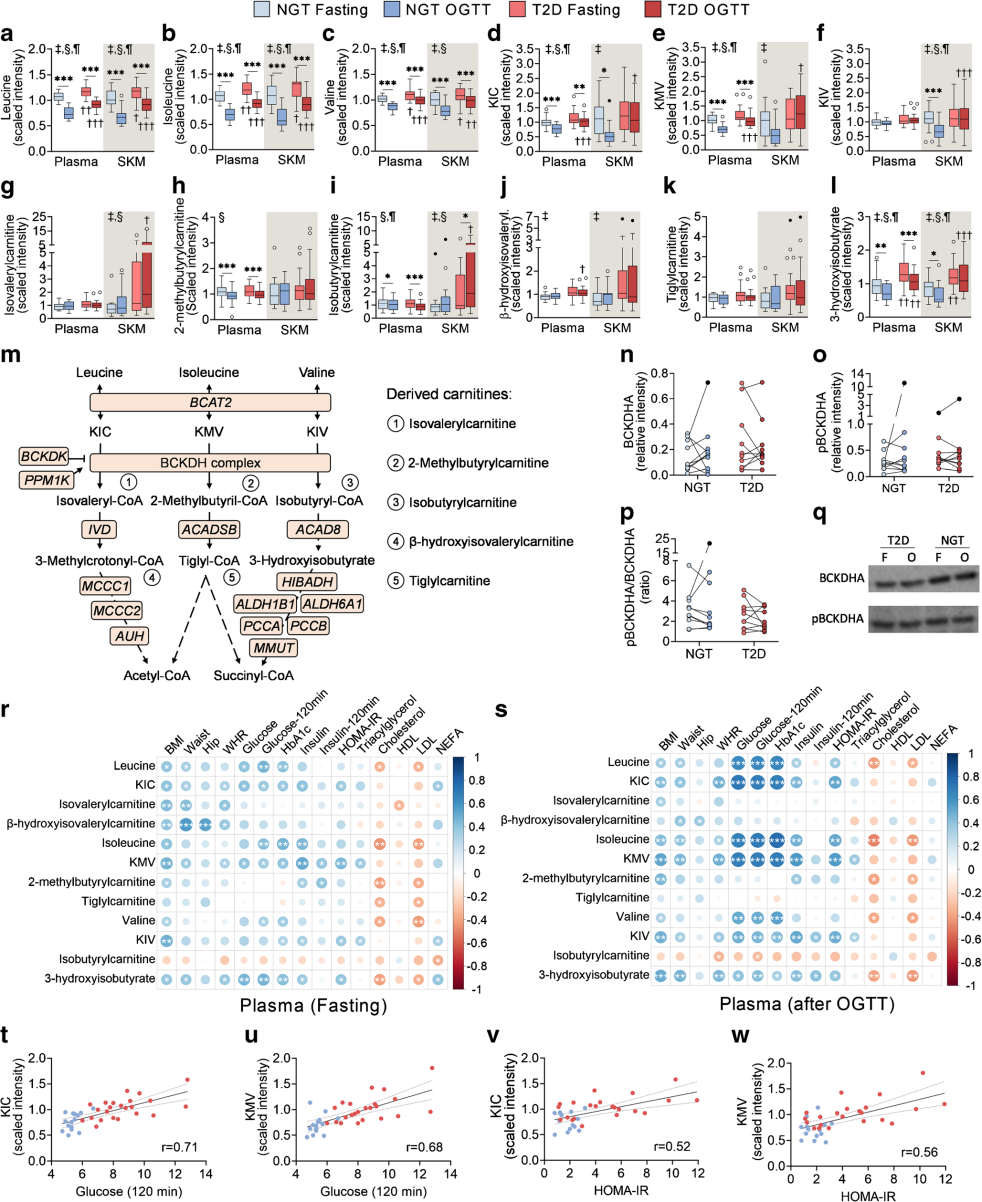
Glucose loading further attenuates BCAA catabolism in individuals with type 2 diabetes. (**a**–**l**) Boxplots show scaled intensity values of BCAAs (**a**–**c**), BCKAs (**d**–**f**), BCAA-derived carnitines (**g**–**k**) and 3-hydroxyisobutyrate (**l**) in plasma and skeletal muscle from individuals with NGT (*n* = 15) or type 2 diabetes (*n* = 26), before (fasting) and after a 120 min OGTT. (**m**) Catabolic pathway of BCAAs and genes encoding involved enzymes (in orange boxes). BCKDH complex subunits are encoded by *BCKDHA*, *BCKDHB*, *DBT* and *DLD*. Dashed arrows indicate intermediate metabolites (not shown). (**n**–**p**) Western blot analysis of BCKDHA and pBCKDHA (*n* = 10 per group). Expression values are expressed as relative to a control sample. Black circles represent outlier values. (**q**) Representative immunoblots of BCKDHA and pBCKDHA in fasting conditions (F) and after an OGTT (O). (**r**, **s**) Spearman correlation coefficients between plasma BCAA metabolites and clinical variables. Colour and size are proportional to correlation strength. (**t**–**w**) Examples of Spearman correlation coefficients summarised in (**r**, **s**). Statistical analysis was performed using two-way mixed-design ANOVA followed by Sidak’s post hoc test. *p < 0.05, **p < 0.01 and ***p < 0.001; ^†^*p* < 0.05, ^††^*p* < 0.01 and ^†††^*p* < 0.001 vs NGT at the same feeding state. ‡, condition effect; §, glucose loading effect; ¶, interaction effect. Main and interaction effects symbols indicate *p* < 0.05 to *p* < 0.0001. See also ESM Fig. 1. β-Hydroxyisovaleryl, β-hydroxyisovalerylcarnitine; KIC, α-ketoisocaproate; KIV, keto-isovaline; KMV, keto-methylvalerate, SKM, skeletal muscle; T2D, type 2 diabetes

Metabolomic profiling reveals that a glucose ingestion in fasted individuals elicits an insulin-dependent metabolic response, which is blunted in individuals with impaired glucose tolerance [23]. To assess whether this impaired response also affects the BCAA profile, we measured levels of leucine, isoleucine, valine and derived metabolites in plasma and vastus lateralis biopsies from individuals with either NGT or type 2 diabetes before and after an OGTT. Under fasted conditions, the three BCAAs were ~10% and ~13% higher in plasma and skeletal muscle, respectively, from individuals with type 2 diabetes as compared with NGT (Fig. 2a–c), and non-significant changes were found in the corresponding branched-chain α-keto acids (BCKAs) (Fig. 2d–f). While BCAA-derived acylcarnitines were not different between groups (Fig. 2g–k), fasting 3-hydroxyisobutyrate (3-HIB) was higher in plasma (+37%) and skeletal muscle (+45%) from individuals with type 2 diabetes as compared with NGT (Fig. 2l). This valine-derived metabolite (Fig. 2m) promotes insulin resistance in skeletal muscle cells by increasing fatty acid uptake [24].

Glucose ingestion decreased circulating and intramuscular levels of BCAA and BCKA (Fig. 2a–f and ESM Fig. 1a,b), albeit to a lesser extent in individuals with type 2 diabetes. The intramuscular content of BCKA was decreased 37–56% in individuals with NGT, whereas levels remained unaltered in those with type 2 diabetes. These differences were not driven by the higher BMI and age in the type 2 diabetes group (ESM Table 3). BCKAs are irreversibly decarboxylated by the branched-chain keto acid dehydrogenase (BCKDH) complex, the rate-limiting enzyme in the catabolism of BCAA. We determined total abundance and phosphorylated (inactive) BCKDHA content in skeletal muscle (Fig. 2n–q) and found no differences between groups. Glucose loading decreased 3-HIB levels in skeletal muscle of individuals with NGT but not type 2 diabetes (Fig. 2l). Collectively, these results suggest that a glucose challenge unmasks defects at several steps of BCAA catabolism in type 2 diabetes. Indeed, circulating levels of leucine, isoleucine and derived BCKAs exhibited a positive correlation (*r* = 0.64–0.77) with blood glucose, HbA_1c_ levels and HOMA-IR after an OGTT (Fig. 2r–w and ESM Fig. 1c,d).

### Expression of genes involved in BCAA catabolism is decreased in skeletal muscle from individuals with type 2 diabetes

**Fig. 3:**
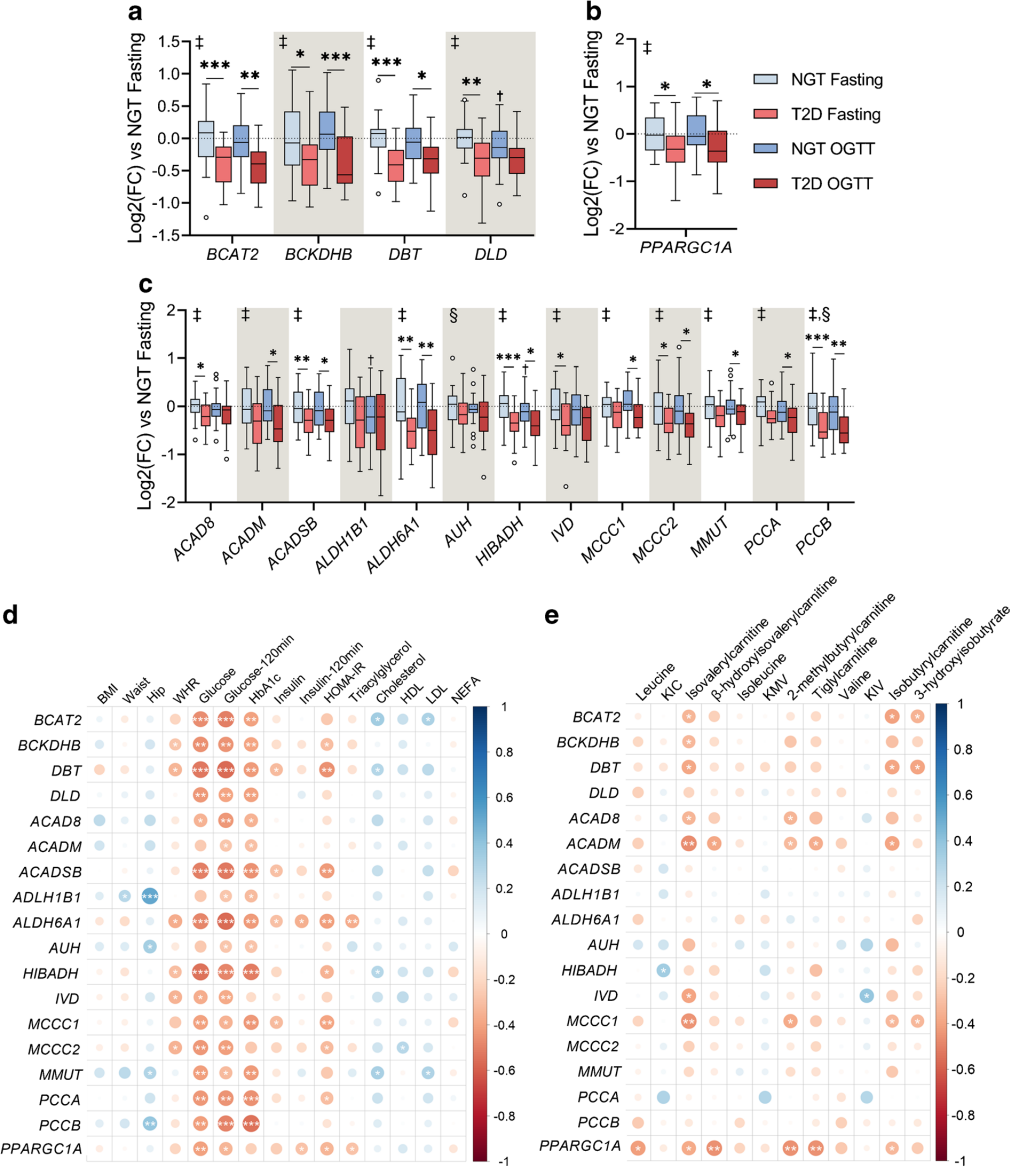
Expression of genes involved in BCAA catabolism in individuals with type 2 diabetes and correlations with blood glucose levels. (**a**, **c**) BCAA gene expression in skeletal muscle biopsies of individuals with NGT (*n* = 25) or type 2 diabetes (*n* = 25) before and after a 120 min OGTT. (**b**) Expression of the transcriptional coactivator *PPARGC1A*. Gene expression is shown as log_2_(fold change) relative to the NGT fasting group. Statistical analysis was performed using two-way mixed-design ANOVA followed by Sidak’s post hoc test. (**d**, **e)** Spearman correlation coefficients between BCAA genes and clinical variables (**d**) or skeletal muscle BCAA metabolites (**e**) in fasted skeletal muscle. Colour and size are proportional to correlation strength. *p < 0.05, **p < 0.01 and ***p < 0.001; ^†^*p* < 0.05 vs NGT fasting. ‡, condition effect; §, glucose loading effect. Main and interaction effects symbols indicate *p* < 0.05 to *p* < 0.0001. See also ESM Fig. 2. FC, fold change; KIC, α-ketoisocaproate; KIV, keto-isovaline; KMV, keto-methylvalerate; T2D, type 2 diabetes

Expression of genes encoding enzymes involved in the first steps of BCAA metabolism, namely branched-chain amino acid transaminase 2 (BCAT2) and three subunits of the BCKDH complex were decreased in skeletal muscle of individuals with type 2 diabetes (Fig. 3a). Furthermore, 69% of the analysed genes participating in metabolic steps downstream of BCKDH showed a similar profile (Fig. 3c), indicating that BCAA gene expression is widely downregulated in skeletal muscle of individuals with type 2 diabetes. These differences were also evident after an OGTT, as mRNA levels remained relatively stable. PGC-1α is an upstream regulator of BCAA metabolism [15]. Expression of *PPARGC1A*, which encodes PGC-1α, was decreased in skeletal muscle of individuals with type 2 diabetes, irrespective of the feeding status (Fig. 3b). In addition, *PPARGC1A* was positively correlated with BCAA gene expression (ESM Fig. 2a) and BCAA metabolites in individuals with NGT but not type 2 diabetes (ESM Fig. 2b,c), while the expression of several BCAA genes was inversely associated with blood glucose and HbA_1c_ (Fig. 3d). Expression of BCAA genes did not consistently correlate with BCAA-related metabolites (Fig. 3e) but exhibited opposite patterns in individuals with NGT and type 2 diabetes (ESM Fig. 2b,c).

### PGC-1α mediates BCAA gene expression in primary HSMCs

**Fig. 4:**
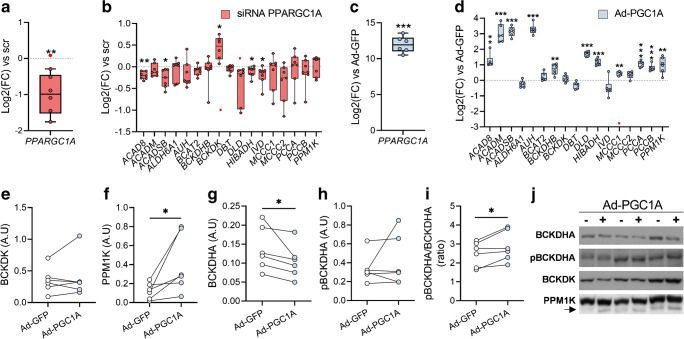
PGC-1α mediates BCAA gene expression in HSMCs. (**a**) Efficiency of *PPARGC1A* siRNA-mediated silencing. (**b**) Effects of the silencing of *PPARGC1A* on the expression of BCAA catabolic genes, *BCKDK* and *PPM1K*. (**c**) Expression of *PPARGC1A* in Ad-PGC1A cells. (**d**) Effects of Ad-PGC1A on the expression of BCAA catabolic genes, *BCKDK* and *PPM1K*. Gene expression is shown as log_2_(fold change) relative to the corresponding scramble-treated cells (dotted line). (**e**–**i**) Protein levels of BCKDK (**e**), PPM1K (**f**), BCKDHA (**g**) and phosphorylated BCKDHA (**h**), and the pBCKDHA/BCKDHA ratio (**i**). (**j**) Representative immunoblots. Arrow indicates the band corresponding to PPM1K protein. Statistical analysis was performed using paired *t* test (*n* = 6). *p < 0.05, **p < 0.01 and ***p < 0.001 vs scr (**a**, **b**) or Ad-GFP (**c**–**i**). Red circles indicate significant outliers not considered in statistical calculations. A.U, arbitrary units; FC, fold change; scr, scrambled siRNA

Primary HSMCs transfected with *PPARGC1A* siRNA displayed reduced expression of *PPARGC1A* (Fig. 4a). This transfection moderately decreased the expression of genes of the family of acyl-CoA dehydrogenases (*ACAD8*, *ACADSB* and *IVD*), and *HIBADH* (encoding the 3-H dehydrogenase enzyme), as compared with cells treated with a scrambled siRNA (Fig. 4b). Adenovirus-mediated *PPARGC1A* overexpression (Ad-PGC1A) in HSMCs (Fig. 4c) increased the expression of 61% of the genes relative to adenovirus-GFP cells (Fig. 4d). We found increased PPM1K (Fig. 4f) and reduced BCKDH protein content in Ad-PGC1A cells, associated with a higher pBCKDHA/BCKDHA ratio (Fig. 4g,i).

### Mice with skeletal muscle-specific modified expression of *Ppargc1a* exhibit altered levels of BCAA gene transcripts and related metabolites

**Fig. 5:**
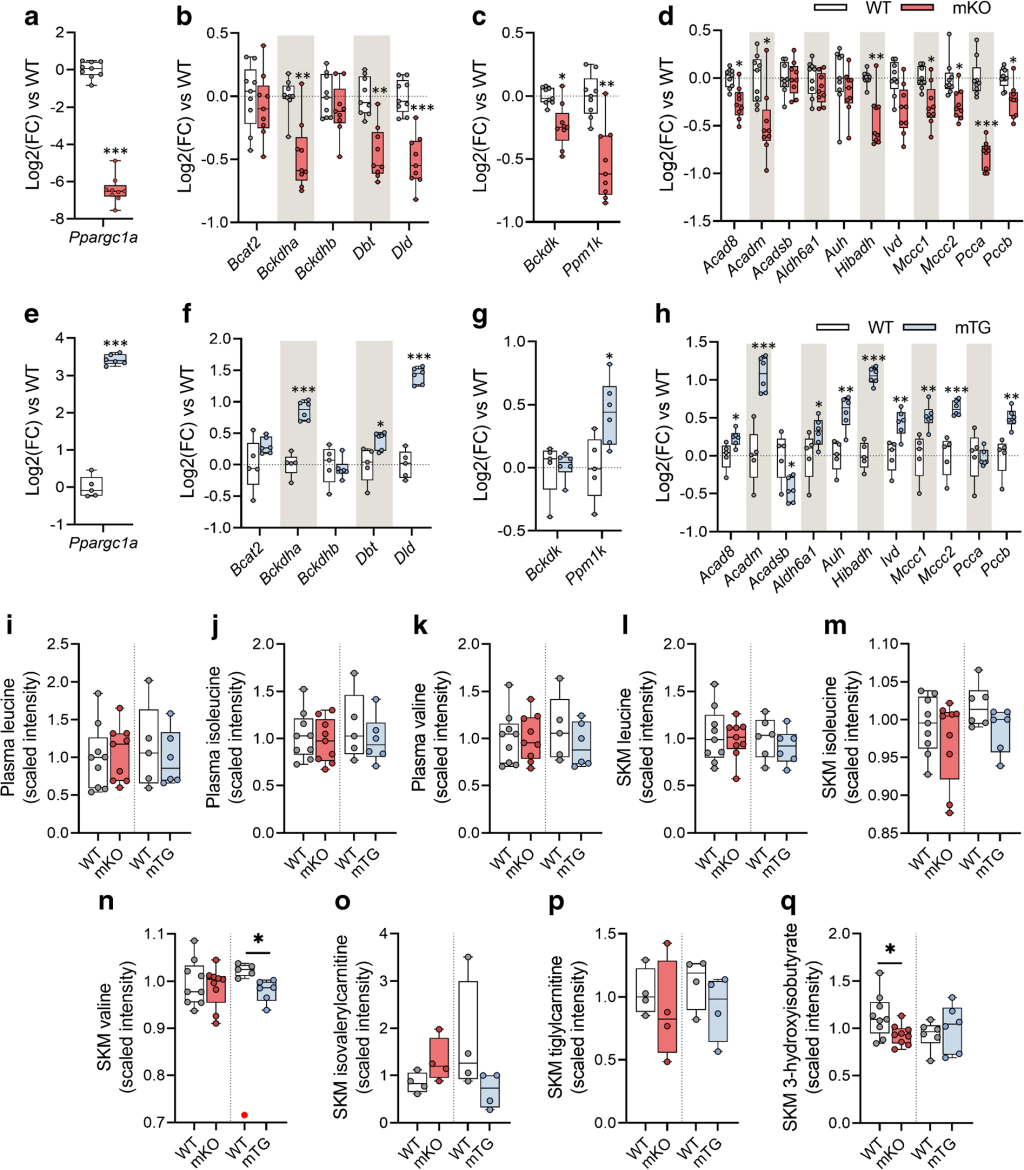
Mice with skeletal muscle-specific modified expression of *Ppargc1a* exhibit altered levels of BCAA gene transcripts and related metabolites. (**a**) Quadriceps *Ppargc1a* expression in mKO mice and corresponding littermates (*n* = 9). (**b**–**d**) Expression of genes encoding BCAT2 and BCKDH subunits (**b**), BCKDK and PPM1K (**c**) and BCAA catabolic enzymes (**d**) in mKO mice. (**e**) Quadriceps *Ppargc1a* expression in mTG mice (*n* = 6) and corresponding littermates (*n* = 5). (**f**–**h**) Expression of genes encoding BCAT2 and BCKDH subunits (**f**), BCKDK and PPM1K (**g**) and BCAA catabolic enzymes (**h**) in mTG mice. (**i**–**k**) Scaled intensity values of leucine, isoleucine and valine in plasma from mKO mice (*n* = 9), mTG mice (*n* = 6) and corresponding WT littermates (*n* = 9 and *n* = 5, respectively). (**l**–**q**) Scaled intensity values of BCAA and downstream intermediate metabolites in skeletal muscle from mKO mice (*n* = 9), mTG mice (*n* = 6) and corresponding WT littermates (*n* = 9 and *n* = 5, respectively). Isovalerylcarnitine and tiglylcarnitine were measured in a subset of animals from each group (*n* = 4). Gene expression is shown as log_2_(fold change) relative to WT mice. The dotted line represents the mean of the WT group. Statistical analysis was performed using unpaired *t* test. *p < 0.05, **p < 0.01 and ***p < 0.001 vs WT. Red circles indicate significant outliers not considered in statistical calculations. See also ESM Fig. 3. FC, fold change; SKM, skeletal muscle

Muscle-specific *Ppargc1a* (encoding PGC-1α) knockout mice (mKO) had normal body weight (ESM Fig. 3a) and slightly impaired glucose tolerance (ESM Fig. 3b,c) as compared with wild-type (WT) littermates. Skeletal muscle from mKO mice (Fig. 5a) displayed decreased expression of most (67%) of the analysed BCAA genes relative to WT littermates (Fig. 5b–d). Accordingly, a contrasting profile of BCAA gene expression was observed in skeletal muscle-specific *Ppargc1a* transgenic mice (mTg) vs the mKO mice, with an upregulation of the BCAA genes relative to WT littermates (Fig. 5e–h). These changes in gene expression were not associated with alterations in either body weight or glucose tolerance (ESM Fig. 3d–f).

To ascertain whether PGC-1α-associated alterations in BCAA gene expression have functional implications in BCAA metabolism, we performed LC/MS metabolomics to profile BCAA-related metabolites in plasma and quadriceps muscle from mKO and mTG mice. Circulating BCAA levels in mKO and mTG mice were unaltered relative to respective WT littermate control mice (Fig. 5i–k), whereas the muscle content of valine was reduced in mTG mice (Fig. 5l–n). Similar non-significant changes were observed for isoleucine (*p* = 0.09) and isovalerylcarnitine (*p* = 0.12) (Fig. 5m,o), while mKO mice exhibited decreased levels of 3-HIB (Fig. 5q). Consistent with our results in HSMCs, C2C12 Ad-PGC1A upregulated the expression of BCAA-related genes (ESM Fig. 3g), and this was associated with increased leucine oxidation (ESM Fig. 3h).

### PGC-1α regulates BCAA gene transcription through ERRα

**Fig. 6:**
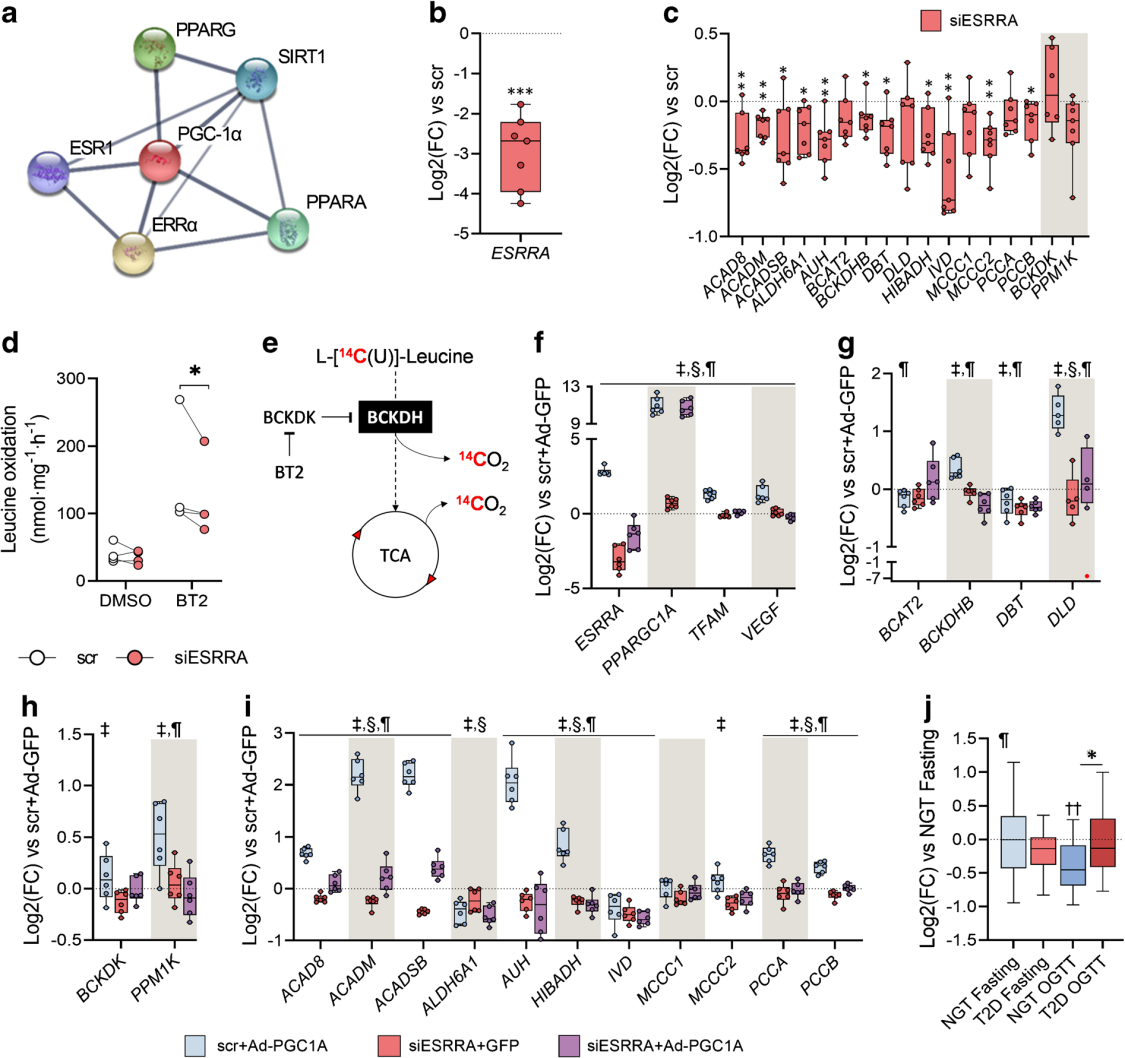
PGC-1α regulates BCAA gene transcription through ERRα. (**a**) STRING Interacting Network showing the top five interacting proteins for PGC-1α. (**b**) Efficiency of *ESRRA* silencing in primary HSMCs. (**c**) Expression of BCAA genes. (**d**) Leucine oxidation rates in HSMCs. (**e**) Schematic biochemical representation of the leucine oxidation assay. (**f**) Expression of *ESRRA*, *PPARGC1A* and target genes in Ad-PGC1A cells transfected with *ESRRA* siRNA. (**g–i**) BCAA gene expression in Ad-PGC1A cells with or without *ESRRA* siRNA. (**j**) Expression of *ESRRA* in skeletal muscle biopsies from individuals with NGT or type 2 diabetes. Gene expression is shown as log_2_(fold change) relative to the corresponding scramble+GFP treated cells (dotted line), scr (**b**, **c**), scr + Ad-GFP (**f**–**i**) or NGT fasting (**j**). Statistical analysis was performed using paired *t* test (**b**, **c**, *n* = 7), two-way repeated measures ANOVA followed by Tukey’s post hoc test (**d**, *n* = 4; **f**–**i**, *n* = 6, except in **g**: *DLD*, *n* = 5), or two-way mixed-design ANOVA followed by Sidak’s post hoc test (**j**, *n* = 25). *p < 0.05, **p < 0.01 and ***p < 0.001 vs scr (**b**, **c**) or scr-Ad-GFP (**f**–**i**). ‡, siESRRA effect; §, Ad-PGC1A effect; ¶, interaction effect. Main and interaction effects symbols indicate *p* < 0.05 to *p* < 0.0001. See also ESM Fig. 4.; BT2, 3,6-dichlorobrenzo(b)thiophene-2-carboxylic acid; ESR1, oestrogen receptor 1; FC, fold change; scr, scrambled siRNA; SIRT1, sirtuin 1; TCA, tricarboxylic acid cycle

The orphan nuclear receptor ERRα, encoded by *ESRRA*, is a canonical functional partner of PGC-1α (Fig. 6a) that regulates metabolic processes in mitochondria [25]. Thus, we investigated whether ERRα is necessary for PGC-1α-enhanced BCAA gene expression. siRNA-mediated silencing of ERRα (Fig. 6b) decreased the expression of 65% of the analysed genes (Fig. 6c). We determined whether this BCAA gene downregulation affects leucine oxidation in skeletal muscle. HSMCs were treated with either DMSO or BT2, a BCKDK inhibitor that increases BCAA oxidative flux. *ESRRA* silencing decreased leucine oxidation rates under BCKDH-activated conditions, as measured by CO_2_ production after incubation with [U-^14^C]leucine (Fig. 6d,e). We next tested whether ERRα is a PGC-1α interacting partner in the transcriptional regulation of the BCAA gene set using HSMCs treated with either scramble siRNA or *ESRRA* siRNA and transfected with Ad-GFP or Ad-PGC1A. We also tested this using Ad-PGC-1A cells treated with the inverse ERRα agonist XCT-790 (ESM Fig. 4). Since *PPARGC1A* and *ESRRA* mutually regulate their expression, we confirmed that *ESRRA* silencing did not abrogate *PPARGC1A* in cultured cells. Ad-PGC1A cells had high levels of *PPARGC1A* transcripts regardless the treatment with *ESRRA* siRNA (Fig. 6f and ESM Fig. 4a), whereas the expression of two known targets of PGC-1α/ERRα, *TFAM* and *VEGF*, was dampened by *ESRRA* siRNA despite the overexpression of *PPARGC1A*. Knockdown and inhibition of *ESRRA* completely ablated PGC-1α-mediated upregulation in all analysed genes (Fig. 6g–i and ESM Fig. 4b–d), indicating that ERRα is essential for the transcriptomic regulation of the BCAA gene network orchestrated by PGC-1α. Nevertheless, *ESRRA* expression was similar between fasted individuals with either NGT or type 2 diabetes (Fig. 6j).

## Discussion

A link between increased plasma BCAAs and insulin resistance was established as early as 1969 [3] but this association has remained relatively unexplored until the last decade. Here, we elucidate the mechanisms underpinning BCAA metabolism in type 2 diabetes. While we corroborate the association between BCAA metabolites and type 2 diabetes [9, 26–28] and the role of PGC-1α in BCAA catabolism [15, 24, 29], we provide new evidence that an OGTT unmasks impairments in BCAA catabolism in individuals with type 2 diabetes. Moreover, we reveal that PGC-1α-mediated regulation of genes important for BCAA catabolism is dependent on ERRα, a canonical PGC-1α-interacting protein.

Circulating plasma BCAA levels are altered in individuals with type 2 diabetes; however, metabolomic profiles of peripheral tissues involved in glucose homeostasis are scarce and the transcriptomic regulation of genes involved in BCAA catabolism is unknown. Studies focusing on skeletal muscle BCAA content are limited to an analysis of adults with insulin resistance [14], rather than type 2 diabetes. We performed untargeted metabolomic analysis on both plasma and vastus lateralis biopsies obtained before and after an OGTT from men with either NGT or type 2 diabetes. Corroborating an earlier study of plasma samples from the Framingham Heart Study Offspring cohort [30], circulating BCAAs were decreased after an OGTT and this reduction was attenuated in type 2 diabetes. Concomitantly, we found that this blunted response also affected leucine- and isoleucine-derived BCKAs. In skeletal muscle, insulin inhibits proteolysis [31] and increases the preference for BCAA oxidation [13], which may explain the blunted excursion of BCAA and BCKA metabolites in insulin-resistant individuals with type 2 diabetes as compared with NGT. Consistent with this hypothesis, BCAAs, BCKAs and 3-HIB levels after the glucose challenge were significantly less decreased in the skeletal muscle from individuals with type 2 diabetes. Although we did not detect changes in BCKDHA phosphorylation state, BCAA catabolic flux cannot be predicted by BCKDH phosphorylation status [13], thus, we cannot exclude the possibility that BCKDH activity per se is impaired. Moreover, insulin modulates BCAA transamination in type 2 diabetes [32] and BCAT2 activity [33], suggesting that insulin resistance could lead to an accumulation of BCKAs due to defects in both BCAA transamination and oxidation.

Generally, plasma metabolites mirrored the skeletal muscle BCAA profile. However, the intramuscular accumulation of isovalerylcarnitine, isobutyrylcarnitine and 3-HIB was not reflected in plasma, suggesting defects in processes controlling the export of metabolites from myocytes in type 2 diabetes. Accordingly, the accumulation of 3-HIB after an OGTT is notable since this valine-derived metabolite promotes insulin resistance through increased fatty acid uptake in skeletal muscle [24]. This could lead to a vicious cycle in which secretion of 3-HIB from skeletal muscle may decrease insulin sensitivity and further impair insulin signalling. Although elevated levels of circulating C3 and C5 acylcarnitines have been also detected in individuals with obesity [7], whether accumulation of short-chain acylcarnitines mediates in insulin resistance remains to be elucidated [34]. These results suggest that the degradation of BCAA-derived metabolites is incomplete. Additionally, we found a strong correlation of post-OGTT BCAAs and derived BCKA circulating levels with blood glucose and HbA_1c_. Since plasma fasting BCAA levels exhibited a much weaker relationship with clinical biomarkers of type 2 diabetes, this finding underscores a tight connection between impaired glucose homeostasis and whole-body BCAA catabolism.

The accumulation of BCAA metabolites in individuals with type 2 diabetes was accompanied by reduced expression of genes involved in BCAA catabolism, suggesting that alterations in the transcriptional regulation of these genes could attenuate BCAA catabolism in skeletal muscle. Contrasting with previous findings [35], we did not find a correlation between BCAA genes and related metabolites, suggesting that post-transcriptional modifications play a key role in the impairment of BCAA catabolism. Conversely, the BCAA gene set was inversely correlated with blood glucose levels, suggesting a connection between glucose homeostasis and alterations in the transcriptional regulation of genes involved in BCAA catabolism.

PGC-1α mediates the expression of genes involved in BCAA catabolism [15, 24, 29]. Concordantly, expression of *PPARGC1A* was positively correlated with BCAA genes. PGC-1α also coordinates metabolic and transcriptomic programs linked to cellular energy homeostasis [36–38], and reduced PGC-1α mRNA and protein levels are linked to insulin resistance in type 2 diabetes [39, 40]. In the present study, primary HSMCs and mouse skeletal muscle with reduced *PPARGC1A* expression exhibited a mild reduction in expression of several BCAA genes, whereas *PPARGC1A* overexpression was associated with a consistent upregulation. Thus, while PGC-1α was not essential for basal BCAA gene transcription, a role in the adaptive response to increased BCAA catabolic demands, such as during exercise or nutritional changes, cannot be excluded [41]. We next hypothesised that alterations in the BCAA gene network would impact BCAA metabolism. We found that mice overexpressing *Ppargc1a* in skeletal muscle exhibited an upregulation of BCAA genes and reduced intramuscular accumulation of valine, suggestive of increased BCAA catabolic flux. Similarly, adenovirus-mediated overexpression of *Ppargc1a* in C2C12 myotubes increased leucine oxidation rates. However, intramuscular levels of BCAA metabolites were unaltered in mKO mice. Thus, other organs such as adipose tissue may compensate for a putative impairment in BCAA catabolism [42]. Furthermore, a metabolic challenge may be necessary to reveal functional alterations in BCAA metabolism in mKO and mTG mice.

PGC-1α is a transcriptional coactivator and does not directly bind DNA in a sequence-specific manner. The nuclear orphan receptor ERRα is one of the main transcriptional partners of PGC-1α [25], and through this physical interaction, elicits the transcription of genes involved in mitochondrial biogenesis and oxidative phosphorylation [43], lipid metabolism [44] and ketone body homeostasis [45]. We found that *ESRRA* silencing, as well as inhibition of ERRα with an inverse agonist, downregulated BCAA genes and abrogated the PGC-1α-induced responses, indicating that ERRα is necessary for the PGC-1α-mediated transcription of BCAA genes. Moreover, *ESRRA* silencing was associated with a modest decrease in BCKDH-stimulated leucine oxidation, suggesting a functional impact of the transcriptional downregulation of BCAA genes under energy-demanding conditions. Thus, we next hypothesised that alterations in ERRα may explain the defects in BCAA catabolism in the individuals with type 2 diabetes. However, expression of *ESRRA* was unaltered in skeletal muscle of individuals with NGT or type 2 diabetes, suggesting that reduction in PGC-1α is sufficient to impair BCAA expression or, alternatively, the PGC-1α–ERRα interaction is compromised in type 2 diabetes.

Some limitations of our study warrant discussion. Our analysis was confined to male participants and therefore we cannot exclude sex-specific differences in the analysed outcomes. Most of the individuals with type 2 diabetes included in this study were treated with metformin. Considering that mitochondria are the major target of metformin, this may affect BCAA metabolism. Due to limitations in the LC/MS methodology, quantification of BCAA metabolites with CoA moieties was not possible. To overcome this, we used specific derived carnitines from these metabolites as a proxy. Alterations in PGC-1α expression may influence fibre type distribution [19, 46], which could impact the results. Nevertheless, similar results were obtained in transgenic mice, in vitro primary HSMCs and C2C12 myotubes, suggesting that alterations in BCAA metabolism are unrelated to fibre type.

In conclusion, altered expression of *PPARGC1A* is associated with disturbances in BCAA metabolism in skeletal muscle of individuals with type 2 diabetes. Experimental approaches to reduce *PPARGC1A* levels partially recapitulates the BCAA gene set profile identified in skeletal muscle from individuals with type 2 diabetes, without impacting circulating and intramuscular BCAA levels. Our results indicate that glucose loading exacerbates disturbances in the BCAA profile, revealing that the metabolic inflexibility that characterises type 2 diabetes encompasses BCAA catabolism. Additionally, our data demonstrate that ERRα is essential for PGC-1α-mediated transcriptional regulation of genes involved in BCAA metabolism in primary human myotubes, thereby unravelling a new role for this orphan nuclear receptor.

## Supplementary Information

ESM (PDF 585 kb)

## Data Availability

The datasets generated during and/or analysed during the current study are available from the corresponding author on reasonable request.

## Abbreviations

Ad-PGC1A: Adenoviral vector for human or mouse *PPARGC1A*
Ad-GFP: Adenoviral vector for *gfp*
BCAA: Branched-chain amino acid
BCAT2: Branched-chain amino acid transaminase 2
BCKA: Branched-chain α-keto acid
BCKDH: Branched-chain keto acid dehydrogenase
BCKDHA: Branched-chain α-keto acid dehydrogenase E1 subunit α
BCKDK: Branched-chain keto acid dehydrogenase kinase
BT2: 3,6-Dichlorobenzo(b)thiophene-2-carboxylic acid
ERRα: Oestrogen-related receptor α
3-HIB: 3-Hydroxyisobutyrate
HSMC: Human skeletal muscle cell
mKO: *Ppargc1a* muscle-specific knockout mice
mTG: Skeletal muscle-specific *Ppargc1a* transgenic mice
NGT: Normal glucose tolerance
PGC-1α: Peroxisome proliferator-activated receptor γ coactivator-1α
PPARγ: Peroxisome proliferator-activated receptor γ
PPM1K: Protein phosphatase, Mg^2+^/Mn^2+^-dependent 1 K
siRNA: Small interfering RNA
WT: Wild-type
