# Supplementary material for: Branched-chain amino acid metabolism is regulated by ERRα in primary human myotubes and is further impaired by glucose loading in type 2 diabetes
Source: Diabetologia. 2021 Jun 16;64(9):2077–91. doi: 10.1007/s00125-021-05481-9 (PMC8382616; doi:10.1007/s00125-021-05481-9)
Supplement: Supplementary file 1 — (PDF 585 kb) [file 125_2021_5481_MOESM1_ESM.pdf]

# Branched-chain amino acid metabolism is regulated by ERRα in primary human myotubes and is further impaired by glucose loading in type 2 diabetes

## Electronic Supplementary Materials (ESM)

## ESM Methods

### Human plasma and skeletal muscle metabolomics

Metabolomic analysis was performed by Metabolon, Inc. (Durham, NC, USA) as described [1]. Equal volume of plasma and equal weights of vastus lateralis biopsy were used for all samples. The protein fraction was removed using a methanol extraction method while allowing maximum recovery of small molecules. The resulting extract was divided into five fractions: one for analysis by ultra-high-performance liquid chromatography-tandem mass spectroscopy (UPLC-MS/MS) with positive ion mode electrospray ionisation, one for UPLC-MS/MS with negative ion mode electrospray ionisation, one for liquid chromatography (LC) polar platform, one for gas chromatography–MS (GC–MS), and one sample was reserved as a backup.

The LC/MS portion of the UPLC-MS/MS platform was based on a Waters ACQUITY UPLC and a Thermo Scientific Q-Exactive high resolution/accurate mass spectrometer interfaced with a heated electrospray ionisation source and Orbitrap mass analyzer operated at 35,000 mass resolution. Separated dedicated columns (Waters UPLC BEH C18-2.1x100 mm, 1.7 µm) were used to perform analysis under acidic positive ion optimised conditions and basic negative ion optimised conditions. The third aliquot was analysed via negative ionisation following elution from a HILIC column (Waters UPLC BEH Amide 2.1x150 mm, 1.7 µm) using a gradient consisting of water and acetonitrile with 10 mmol/l ammonium formate.

Samples destined for GC-MS analysis were dried under vacuum for at least 18 h prior to being derivatised under dried nitrogen using bistrimethyl-silyltrifluoroacetamide. Derivatised samples were separated on a 5% diphenyl / 95% dimethyl polysiloxane fused silica column (20

m x 0.18 mm ID; 0.18 μm film thickness) with helium as carrier gas and a temperature ramp from 60°C to 340°C within a 17.5-min period. Samples were analysed on a Thermo-Finnigan Trace DSQ fast-scanning single-quadrupole mass spectrometer using electron impact ionisation and operated at unit mass resolving power. The scan range was from 50–750 m/z.

Compounds were identified by comparison to library entries of purified standards or recurrent unknown entities. Metabolon maintains a library based on authenticated standards that contains the retention time/index (RI), mass to charge ratio (m/z), and chromatographic data on all molecules present in the library. Biochemical identifications were based on three criteria: retention index within a narrow RI window of the proposed identification, accurate mass match to the library and the MS/MS forward and reverse scores between the experimental data and authentic standards. The MS/MS scores were based on a comparison of the ions present in the experimental spectrum to the ions present in the library spectrum.

**Mouse plasma and skeletal muscle metabolomics**

Mouse sample preparation and analysis was performed at the Swedish Metabolomics Centre (Umeå University). Prior to gas chromatography time-of-flight mass spectrometry (GC-TOF/MS) analysis, a two-step derivatisation procedure was carried out to increase metabolites volatility and reduce number of tautomeric forms. Derivatised samples were analysed on an Agilent 6890 gas chromatograph equipped with a 10 m × 0.18 mm i.d. fused silica capillary column with a chemically bonded 0.18-μm DB 5-MS stationary phase (J&W Scientific, Folsom, CA, USA). The injector temperature was 270°C. The column temperature was held at 70°C for 2 min, increased by 40°C $min^{-1}$ to 320°C, and held there for 1 min. The column effluent was introduced into the ion source of a Pegasus III time-of-flight mass spectrometer, GC-TOF/MS (Leco, St. Joseph, MI, USA). The transfer line and ion source temperatures were

250°C and 200°C, respectively. Ions were generated by a 70-eV electron beam at an ionisation current of 2.0mA. Spectra were recorded in the mass range 50–800 m/z at 30 spectra/s.

Samples destined to liquid chromatography time-of-flight mass spectrometry (LC-TOF/MS) were re-suspended in methanol and water (1:1) and injected onto a Waters Acquity UPLC HSS T3 C18 column (2.1 × 50 mm, 1.8 μm; Waters Corporation, Milford, MA, USA) in combination with a 2.1 mm x 5 mm, 1.8 μm VanGuard precolumn (Waters Corporation) held at 40°C. Metabolites chromatographic separation were carried out using a gradient solvent system consisting of water with 0.1% formic acid and acetonitrile/isopropanol (75/25, v/v) with 0.1% formic acid at a flow rate of 0.5 mL/min. The detection of separated metabolites was performed using the Agilent 6550 Q-TOF mass spectrometer equipped with a jet stream electrospray ionisation source, operating in both positive and negative ion modes. A reference interface was connected for accurate mass measurements; the reference ions purine (4 μM) and HP-0921 (Hexakis (1H, 1H, 3H-tetrafluoropropoxy) phosphazine) (1 μM) were infused directly into the MS at a flow rate of 0.05 mL/min for internal calibration. Full scan MS spectra were collected in a centroid mode over the mass range 70-1700 m/z with an acquisition rate of 4 $spectras^{-1}$. The Auto MS/MS was performed on QC-samples. The isolation width was set to narrow, i.e. approx. 1.3 amu, the mass range was 40-1700, and data was collected in centroid mode with an acquisition rate of 3 scans/s. The collision energy was 10, 20 and 40 ev. Data were acquired with MassHunter Acquisition Software B.07.01.

The processing of GC-TOF/MS data and extraction of putative metabolites was conducted as described [2]. Briefly, an in-house MATLAB script was used for the extraction of putative metabolites by matching the mass spectra and retention indices to in-house mass spectral library at the Swedish Metabolomics Centre and the publicly available Max Planck Institute library in Golm. The processing of LC-TOF/MS data and extraction of putative metabolites and lipids were performed by MassHunter Profinder version B.08.00 in combination with

Qualitative Analysis software version B.07.00, PCDL manager version B.07.00 and Mass Profiler Professional™ 13.0 (all from Agilent Technologies Inc., Santa Clara, CA, USA). Annotation of metabolites were done by matching the retention time (MS and MS-MS spectra) against the in-house metabolite library.

**RNA isolation and relative mRNA expression**

Total RNA from human skeletal muscle biopsies, mouse skeletal muscles and cultured myotubes was extracted using TRIzol according to manufacturer's instructions (Thermo Fisher Scientific). Concentration and extraction quality (A260/A280) of RNA samples were determined by spectrophotometry using a Nanodrop ND-1000 (Thermo Fisher Scientific) and equal amounts of RNA were used for cDNA synthesis. cDNA was synthesised using the High Capacity cDNA Reverse Transcription kit with random primers (4368814, Thermo Fisher Scientific). qRT-PCR was performed using either a ViiA 7 Real-Time PCR 384-well system or a StepOne Plus Real-Time PCR 96-well system (Thermo Fisher Scientific). Gene expression was determined using Fast SYBR™ Green Master Mix (4309155, Thermo Fisher Scientific). Sequences of primers used for the qRT-PCR are reported in ESM Tables 1 and 2. Gene expression in human skeletal muscle biopsies and cultured myotubes was normalised to the geometrical mean of *GUSB*, *RPLP0* and *TBP* expression. For C2C12 cells and mouse quadriceps muscle, gene expression was normalised to the geometrical mean of *B2m*, *Rplp0* and *Tbp* expression. Relative gene expression was calculated by the comparative ΔΔCt method and normalised to control group (in human and mouse samples, NGT Fasting and WT mice, respectively) or control samples (*in vitro* experiments, cells from each donor were normalised against their correspondent siRNA scr-treated counterparts).

**Western blot analysis**

Human skeletal muscle biopsies were homogenised in ice-cold lysis buffer [137 mmol/l NaCl, 2.7 mmol/l KCl, 1 mmol/l $MgCl_2$, 0.5 mmol/l $Na_3VO_4$, 10% (vol/vol) glycerol, 1% (vol/vol) Triton X-100, 20 mmol/l Tris, 10 mmol/l NaF, 1 mmol/l EDTA, 1 mmol/l PMSF, and 1% (vol/vol) protease inhibitor cocktail set 1 (Merck, Germany)] using a TissueLyser II (21 Hz, 45 sec, two times. Qiagen, Germany). Homogenates were rotated for 30 min at 4°C and thereafter subjected to centrifugation at 12000 g for 15 min at 4°C. Cells were lysed in ice-cold homogenisation buffer. Lysates were rotated for 30 min at 4°C and subjected to centrifugation at 12000 g for 15 min at 4°C.

Protein concentration in the supernatants of skeletal muscle homogenates and cell lysates was determined by BCA protein assay kit (Pierce Biotechnology, Rockford, IL, USA). Samples were prepared with Laemmli buffer to equal final protein concentrations, separated on Criterion XT Bis-Tris Gels for SDS-PAGE (Bio-Rad, USA) and transferred to PVDF membranes (Merck). Thereafter, membranes were stained with Ponceau S to verify transfer quality and confirm equal protein loading. After blocking with 7.5% nonfat milk in Tris-buffered saline with Tween (TBST; 10 mmol/l Tris·HCl, 100 mmol/l NaCl, 0.02% Tween 20) for 2 h at room temperature, membranes were incubated overnight at 4°C with primary antibodies against β-actin (A5441, Sigma-Aldrich, RRID: AB_476744), GAPDH (sc-47724, Santa Cruz Biotechnology, RRID: AB_627678), BCKDHA (ab68094, Abcam, RRID: AB_2063871), $p^{S293}$-BCKDHA (ab200577, Abcam, RRID: AB_2687944), BCKDK (sc-374425, Santa Cruz Biotechnology, RRID: AB_10988235), and PPM1K (ab67935, Abcam, RRID: AB_1142303), all at a 1:1000 dilution. Antibodies were validated in HSMCs in which the corresponding encoding gene was silenced with siRNA. Membranes were washed with TBST and incubated with horseradish peroxidase-conjugated secondary antibodies (1:10000 – 1:25000). Proteins were visualised by enhanced chemiluminescence (Amersham ECL Western Blotting Detection

Reagent, UK). Bands of interest were semi-quantified by densitometry (QuantityOne, Bio-Rad).

## References


[1] Evans AM, DeHaven CD, Barrett T, Mitchell M, Milgram E (2009) Integrated, nontargeted ultrahigh performance liquid chromatography/electrospray ionization tandem mass spectrometry platform for the identification and relative quantification of the small-molecule complement of biological systems. Anal Chem 81(16): 6656-6667. 10.1021/ac901536h

[2] Chorell E, Ryberg M, Larsson C, et al. (2016) Plasma metabolomic response to postmenopausal weight loss induced by different diets. Metabolomics 12(5): 1-14. 10.1007/s11306-016-1013-x

## ESM Tables

**ESM Table 1. Forward and reverse sequences of primers used in gene expression analysis of human skeletal muscle.**

| Gene | Forward sequence (5' to 3') | Reverse sequence (5' to 3') |
|---|---|---|
| ***ACAD8*** | CAGGAGGAGAGGAAGGATGC | AGTGTGGGTTCTACTCCTGA |
| ***ACADM*** | ACCAGACCTGTAGTAGCTGC | GAATCAACCTCCCAAGCTGC |
| ***ACADSB*** | AGGCTGCTAAGAAGAAATTTCCT | GTAAATGTTTGCAGGGGAGC |
| ***ALDH1B1*** | AACCAGAACCCAAGCGTGAT | GCTGGTTGTAGGGGATGTCT |
| ***ALDH6A1*** | GAACTGCCATCTTCACCACC | TGGATGCCCTGTTTGCCATA |
| ***AUH*** | TGATATACGAGTAGCAGCTTCCT | CCCTCCTGGTTCTGTTCCA |
| ***B2M*** | TGTCTTTCAGCAAGGACTGG | AGCAAGCAAGCAGAATTTGG |
| ***BCAT2*** | CGCTCCTGTTCGTCATTCTC | CTAACACGGTGGGCCCATA |
| ***BCKDHB*** | GAGAGGTAGCTTCCATGGCA | TGATTTCCGATGCAAAGCCG |
| ***BCKDK*** | TAACGCCCACCATGATGCT | GCCTGGTCCTTGATCGGA |
| ***DBT*** | AAAACAACTGCTGCTCTCCG | TGCTATCAAACTGAGACACTGT |
| ***DLD*** | TGGTGCAGGAGTAATAGGTGT | GCTTTACCACCAGAAGCAGC |
| ***ESRRA*** | ACTATGGTGTGGCATCCTGT | CCGGACAGCTGTACTCGAT |
| ***HIBADH*** | AGCAGTTTTCATGGATGCCC | AACAGCTCCACAGTACACCA |
| ***IVD*** | CACTTCCAGTTGATGCAGGG | AAACACTGAATGCCGTCCAG |
| ***GUSB*** | CAGAGCGAGTATGGAGCAGA | ACTCTCGTCGGTGACTGTTC |
| ***MCCC1*** | GGGAGACTGCACTTACCTGA | TCCAGCTTTGACAAACACCTT |
| ***MCCC2*** | GGAGGTGGCATTATTACAGGC | TGCTCCTCCCGAATCAACTA |
| ***MUT*** | GCCCTCTTTTCCAGACTCCT | GGTATCACCCCTCCACACAT |
| ***PCCA*** | TGATGAAGAGACCAGGGATGG | TCGAGTCTCCGCATCCAAAA |
| ***PCCB*** | CGATCCCAGTGACCGTCTG | TCCAACAGTCCTCCCATTCA |
| ***PPARGC1A*** | TCTGAGTCTGTATGGAGTGACAT | CCAAGTCGTTCACATCTAGTTCA |
| ***PPM1K*** | AAGCCTTTTCGAGTCATGCC | GCTGCCCCAAACTATTCCAA |
| ***TBP*** | AACAACAGCCTGCCACCTTA | GCCATAAGGCATCATTGGAC |
| ***TFAM*** | CTGTCTTGGCAAGTTGTCCA | CAACGCTGGGCAATTCTTCT |
| ***VEGFA*** | CAGAAGGAGGAGGGCAGAAT | CATCAGGGGCACACAGGAT |

**ESM Table 2. Forward and reverse sequences of primers used in gene expression analysis of mouse skeletal muscle.**

| Gene | Forward sequence (5' to 3') | Reverse sequence (5' to 3') |
|---|---|---|
| ***Acad8*** | GGCTACGGCTATCTGAAGGA | CCTGAAGCAGGTTTCGAGAG |
| ***Acadm*** | GCCCAGAGAGCTCTAGACGA | GTTCAACCTTCATCGCCATT |
| ***Acadsb*** | AAGCCTTGCGAGCTTAACAG | TCCAAGGAGACAAGCAGGTT |
| ***Aldh6a1*** | CTTCTGGATGGGCGAAGAAT | TGTTTCTGTCTCCAAAACCACA |
| ***Auh*** | GCAATTAACCAAGGGATGGA | AGCGAGGAGGTCTTTTCTCC |
| ***B2m*** | TTCTGGTGCTTGTCTCACTGA | CAGTATGTTCGGCTTCCCATTC |
| ***Bcat2*** | TACGTCATTCTGTGCCCTGT | CCTCTCTTCTGGGCTTCTCG |
| ***Bckdha*** | GGCCGGATCTCCTTCTACAT | TAGTCCCGGTACATGAGCAC |
| ***Bckdhb*** | GGTAATACCCCGAAGCCCTT | AGGGTTCTACTGGGACCTGT |
| ***Bckdk*** | GCTACATGAAGACAAGCCTGA | GCATGGGAATGAAGGGGAAC |
| ***Dbt*** | CGCGTGGAATCAAACTCTCC | CCCAATGTTGTGAGAAGCCTT |
| ***Dld*** | GGCATGGTGAAGATTCTTGG | GAAGCACCATATTCCAATGC |
| ***Hibadh*** | GGGAGCTGTTTTCATGGACG | CATTCCAGCAACTCTTGGGC |
| ***Ivd*** | TCGATATTGCCTGTGGACGA | GGGGCCAGGTTCTCTTGAA |
| ***Mccc1*** | TCCAGTGAGTGCAGAAGGAG | ACCATCAGAGAGTCTCCAGC |
| ***Mccc2*** | CAACTTACCTCGGCAAGCAG | GTACAGGAGCCCATGACCAC |
| ***Pcca*** | GCATCCAGTTTCTTGGCACA | TCTGCTACCATGTCTCCAGG |
| ***Pccb*** | TCCTGAGCTGGACACAGTTG | TTTTTGCGTAACTGGGCATT |
| ***Ppargc1a*** | TATGGAGTGACATAGAGTGTGCT | CCACTTCAATCCACCCAGAAAG |
| ***Ppm1k*** | ACAAGGATTAAGCTCTACCATGC | GTGCTGTTGTCTTCGGTACC |
| ***Rplp0*** | AGATTCGGGATATGCTGTTGGC | TCGGGTCCTAGACCAGTGTTC |
| ***Tbp*** | CCTTGTACCCTTCACCAATGAC | ACAGCCAAGATTCACGGTAGA |

**ESM Table 3. Y-intercept p-values corresponding to the analysis of covariance comparing individuals with normal glucose tolerance and type 2 diabetes controlling for variation in BMI and age.**

| | BMI | | Age | |
|---|---|---|---|---|
| | **Plasma** | **SKM** | **Plasma** | **SKM** |
| **Leucine** | <0.0001 | 0.0008 | <0.0001 | <0.0001 |
| **Isoleucine** | <0.0001 | 0.0008 | <0.0001 | <0.0001 |
| **Valine** | 0.0013 | 0.0092 | 0.0003 | 0.0006 |
| **α -ketoisocaproate** | 0.0014 | 0.0300 | 0.0002 | 0.0024 |
| **Keto-methylvalerate** | 0.0021 | 0.0175 | 0.0403 | 0.0106 |
| **Keto-isovaline** | 0.1540 | 0.0184 | 0.0431 | 0.0011 |
| **Isovalerylcarnitine** | 0.4343 | 0.0166 | 0.1120 | 0.0199 |
| **2-methylbutyrylcarnitine** | 0.9525 | 0.1697 | 0.1535 | 0.1250 |
| **Isobutyrylcarnitine** | 0.5386 | 0.0244 | 0.4478 | 0.0189 |
| **β-hydroxyisovalerylcarnitine** | 0.3515 | 0.0625 | 0.3134 | 0.0370 |
| **Tiglylcarnitine** | 0.8905 | 0.1711 | 0.5280 | 0.2819 |
| **3-hydroxyisobutyrate** | 0.0132 | 0.0116 | 0.0008 | 0.0006 |

Linear regression between BCAA metabolites and BMI/Age was calculated in the two experimental groups (after OGTT). Obtained slopes were in all cases not statistically different between groups, allowing to compare Y-intercepts of the regression lines. SKM, skeletal muscle.

## ESM Figures

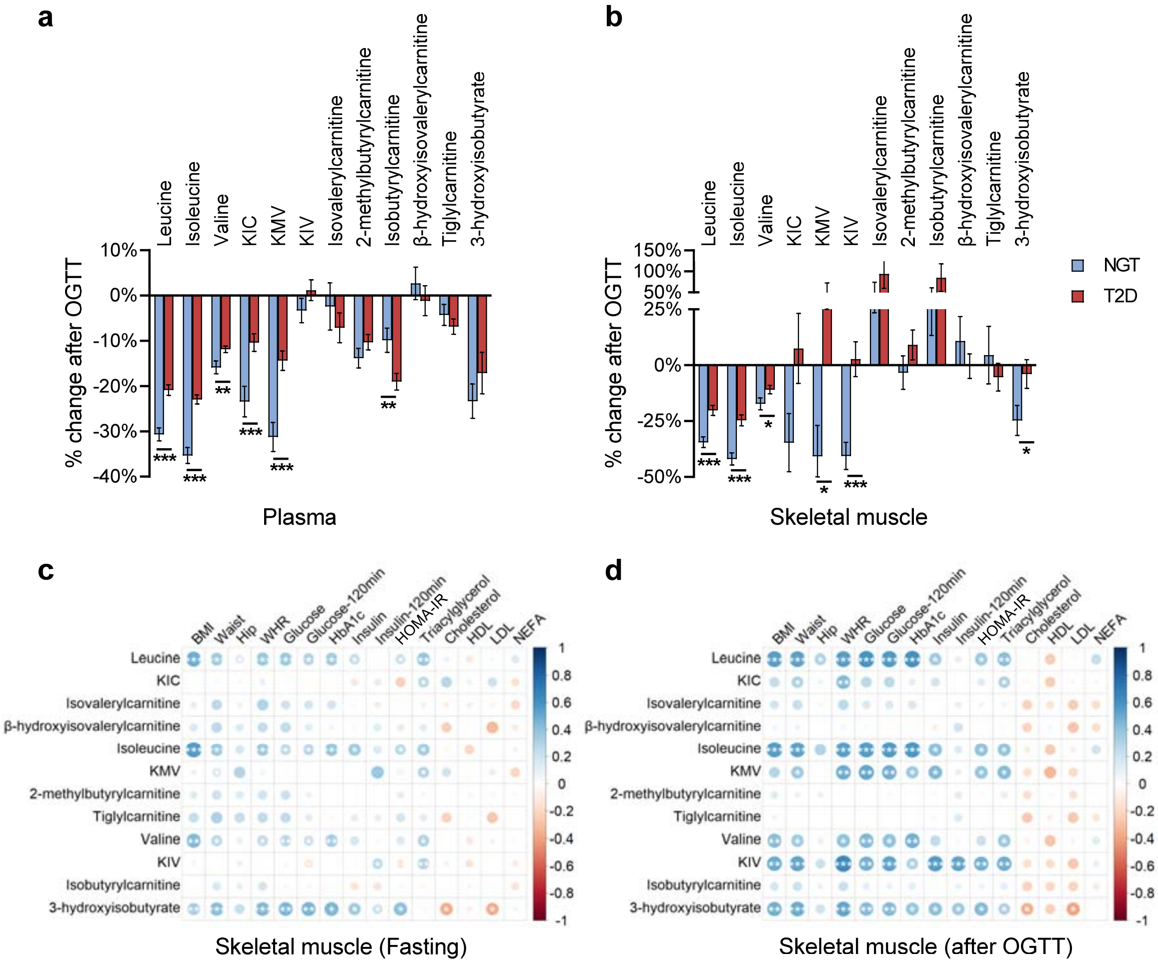


**ESM Figure 1 (Supplementary to Figure 2).** (**a–b**) Metabolite excursions after OGTT in individuals with NGT and T2D. (**c–d**) Spearman correlation coefficients between clinical parameters and skeletal muscle BCAA metabolites measured before (**c**) and after (**d**) an OGTT. Colour and size are proportional to correlation strength. Results are expressed as mean. *, $p$<0.05, **, $p$<0.01 and ***, $p$<0.001. KIC, α-ketoisocaproate; KIV, keto-isovaline; KMV, keto-methylvalerate; T2D, type 2 diabetes.

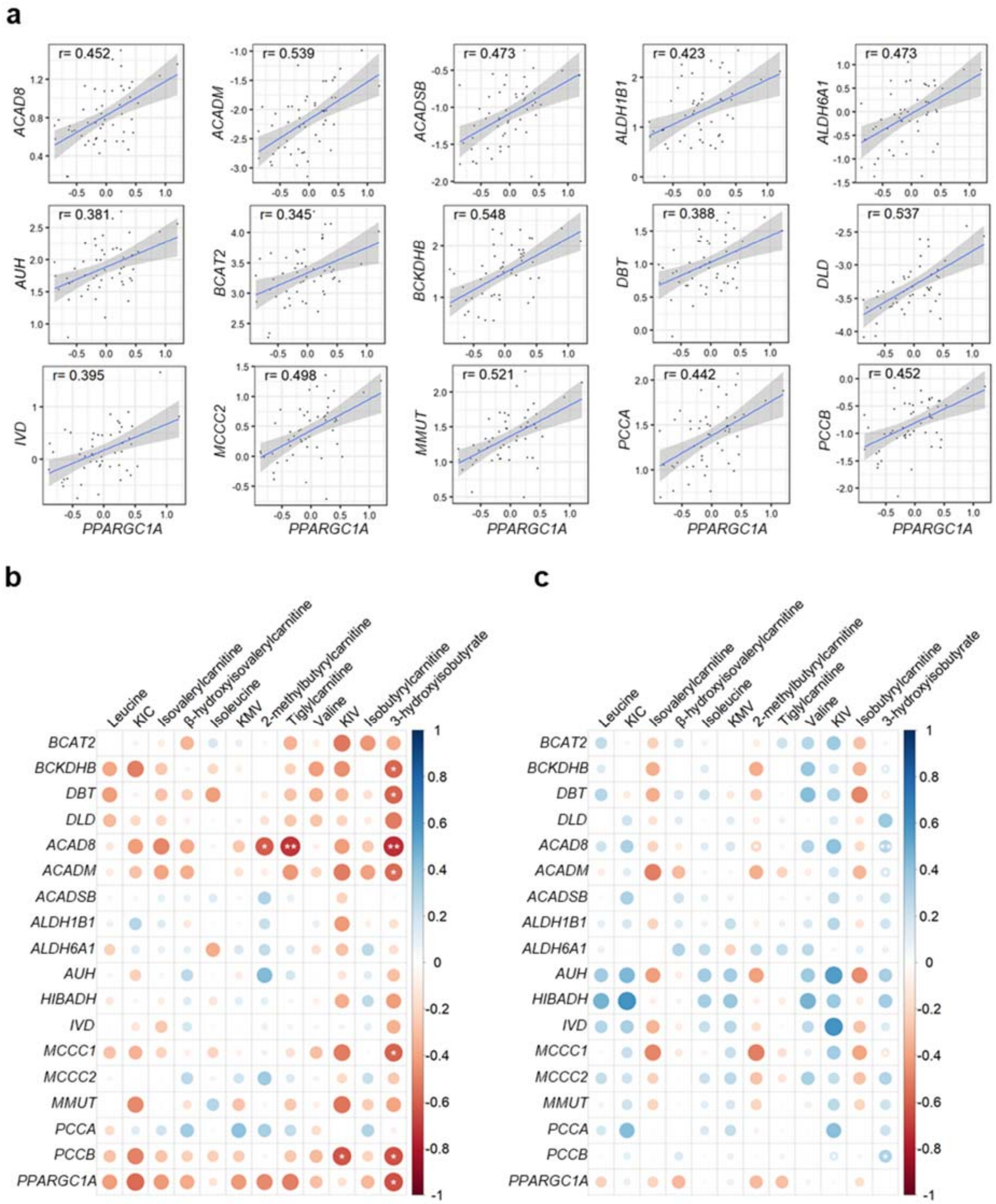


**ESM Figure 2 (Supplementary to Figure 3).** (**a**) Spearman correlation between the skeletal muscle expression of *PPARGC1A* and BCAA genes. (**b–c**) Spearman correlation coefficients between expression of genes involved in BCAA catabolism and skeletal muscle BCAA metabolites measured in individuals with normal glucose tolerance (**b**) and with type 2 diabetes (**c**). Colour and size are proportional to correlation strength; *, $p$<0.05, **, $p$<0.01 and ***, $p$<0.001. KIC, α-ketoisocaproate; KIV, keto-isovaline; KMV, keto-methylvalerate.

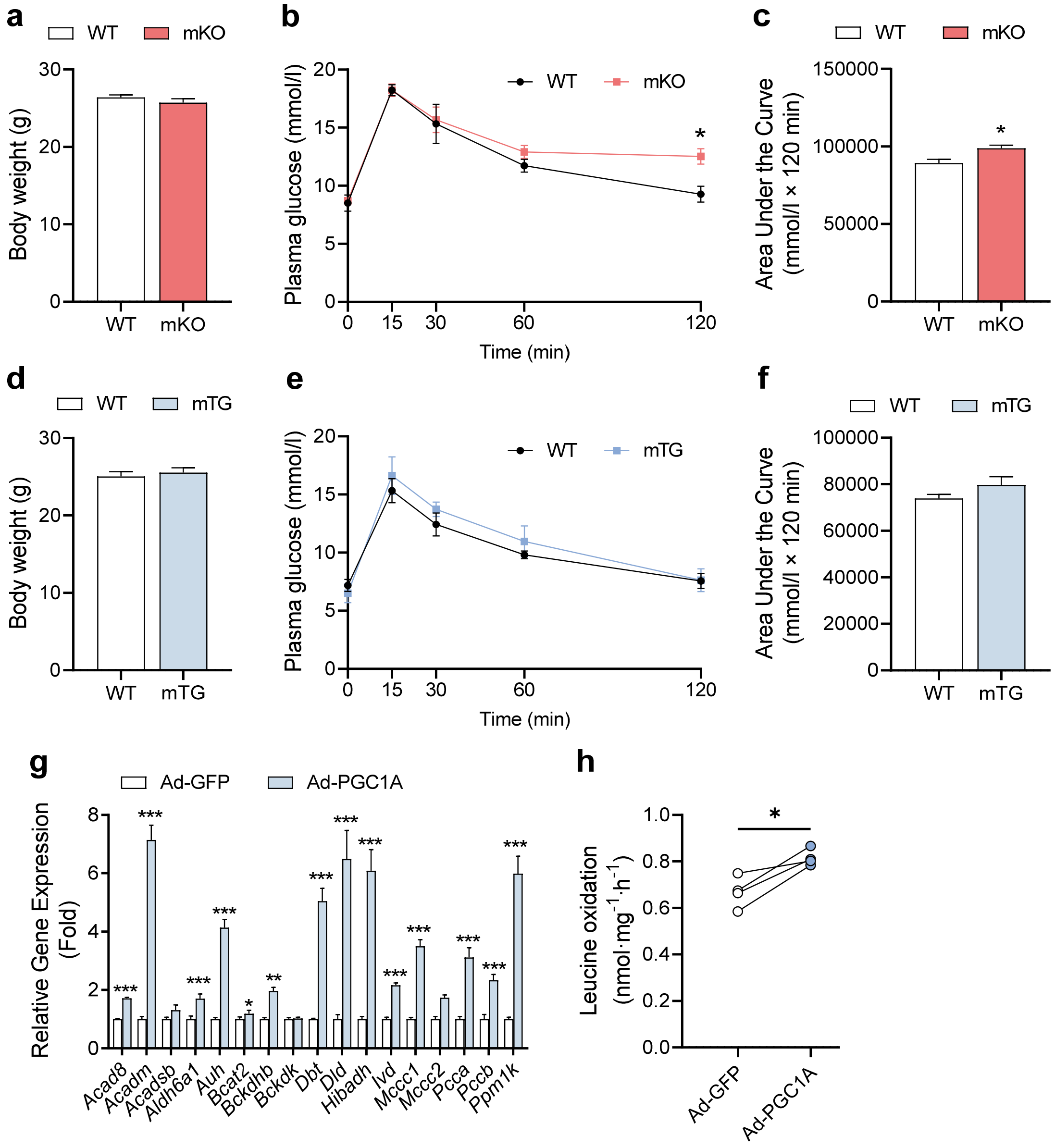


**ESM Figure 3 (Supplementary to Figure 5). Mouse glucose tolerance and BCAA gene expression in *Ppargc1a* overexpressing C2C12 myotubes.** (**a**) Body weight of mKO mice and WT littermates (*n*=9). (**b–c**) Oral glucose tolerance test and corresponding area under the curve of mKO animals and WT littermates (*n*=4). (**d**) Body weight of mTG mice and WT littermates (*n*=6). (**e–f**) Oral glucose tolerance test and corresponding area under the curve of mTG mice and WT littermates (*n*=4). (**g**) BCAA gene expression in Ad-PGC1A C2C12 myotubes (*n*=4). (**h**) Leucine oxidation in Ad-PGC1A C2C12 myotubes (*n*=4). *, $p<0.05$; **, $p<0.01$ and ***$p<0.001$. Results are expressed as mean ± SEM. Ad-GFP, adenoviral overexpression of green fluorescent protein; Ad-PGC1A, adenoviral overexpression of *Ppargc1a*.

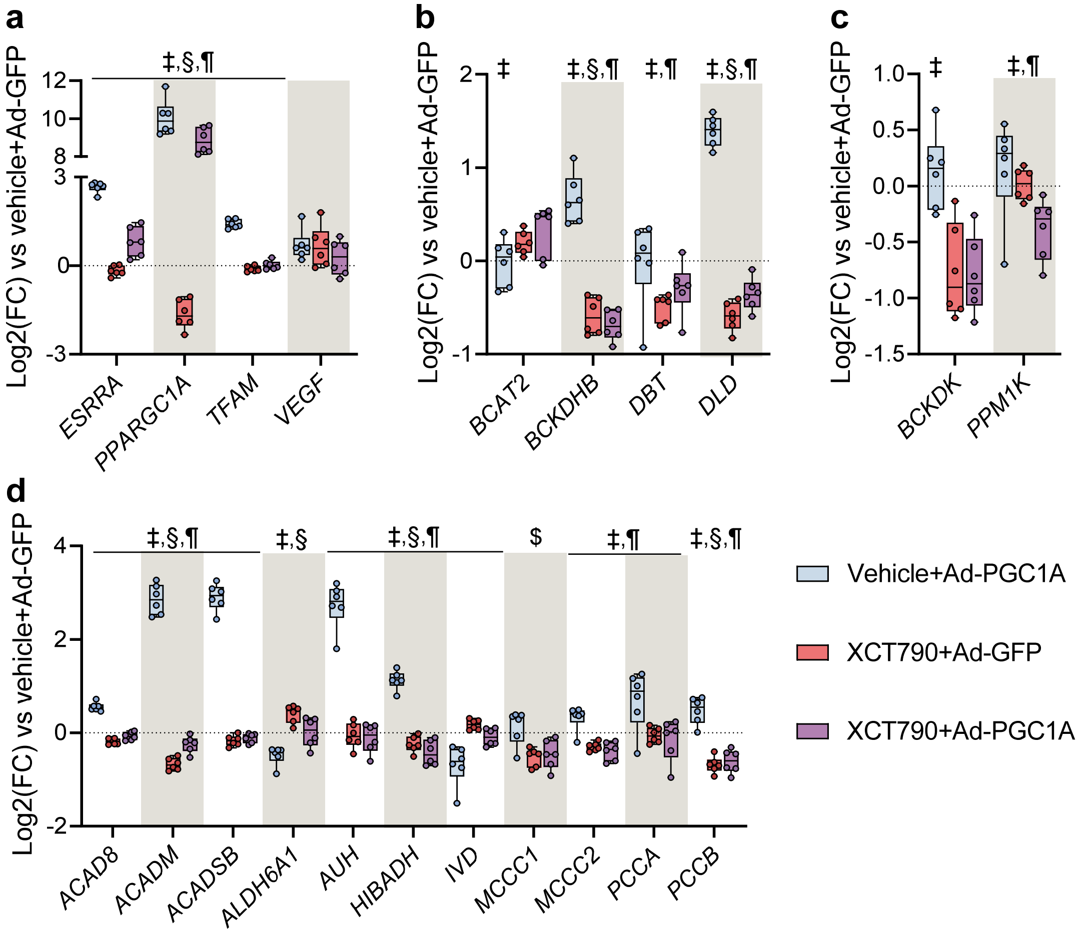


**ESM Figure 4 (Supplementary to Figure 6). Effects of ERRα inverse agonist on BCAA gene expression.** (**a**) Expression of *ESRRA, PPARGC1A* and target genes in Ad-PGC1A cells treated with the ERRα inverse agonist XCT-790. (**b–d**) BCAA gene expression in Ad-PGC1A cells treated with vehicle or XCT-790. Gene expression is shown as log2(fold-change) normalised to the scr+Ad-GFP (dotted line). Statistical analysis was performed using two-way repeated measures ANOVA followed by Tukey's *post-hoc* test ($n$=6). ‡, XCT-790 effect; §, Ad-PGC1A effect; ¶, interaction effect. Main and interaction effects statistical symbols indicate p<0.05 to 0.0001. siRNA, small interfering RNA; scr, scrambled siRNA; Ad-GFP, adenoviral overexpression of green fluorescent protein; Ad-PGC1A, adenoviral overexpression of *PPARGC1A*.
